# Emphatic visualization of sphingomyelin-rich domains by inter-lipid FRET imaging using fluorescent sphingomyelins

**DOI:** 10.1038/s41598-017-16361-x

**Published:** 2017-12-01

**Authors:** Masanao Kinoshita, Hikaru Ano, Michio Murata, Kenta Shigetomi, Junichi Ikenouchi, Nobuaki Matsumori

**Affiliations:** 1 JST-ERATO Lipid Active Structure Project, Osaka University, 1-1 Machikaneyama, Toyonaka, Osaka, 560-0043 Japan; 2 Project Research Center for Fundamental Science, Osaka University, 1-1 Machikaneyama, Toyonaka, Osaka, 560-0043 Japan; 3 Department of Chemistry, Faculty of Science, Kyushu University, 744 Motooka, Nishi-ku, Fukuoka, 819-0395 Japan; 4 Department of Chemistry, Graduate School of Science, Osaka University, 1-1 Machikaneyama, Toyonaka, Osaka, 560-0043 Japan; 5 Department of Biology, Faculty of Science, Kyushu University, 744 Motooka, Nishi-ku, Fukuoka, 819-0395 Japan

## Abstract

Imaging the distribution of sphingomyelin (SM) in membranes is an important issue in lipid-raft research. Recently we developed novel fluorescent SM analogs that exhibit partition and dynamic behaviors similar to native SM, and succeeded in visualizing lateral domain-segregation between SM-rich liquid-ordered (L_o_) and SM-poor liquid-disordered (L_d_) domains. However, because the fluorescent contrast between these two domains depends directly on their partition ratio for the fluorescent SMs, domain-separation becomes indeterminate when the distribution difference is not great enough. In this study, we propose the use of inter-lipid Förster resonance energy transfer (FRET) imaging between fluorescent SMs to enhance the contrast of the two domains in cases in which the inter-domain difference in SM distribution is inadequate for conventional monochromic imaging. Our results demonstrate that inter-lipid FRET intensity was significantly higher in the L_o_ domain than in the L_d_ domain, resulting in a clear and distinguishable contrast between the two domains even in poorly phase-separated giant unilamellar vesicles. In addition, we show that inter-lipid FRET imaging is useful for selective visualization of highly condensed assemblies and/or clusters of SM molecules in living cell membranes. Thus, the inter-lipid FRET imaging technique can selectively emphasize the SM-condensed domains in both artificial and biological membranes.

## Introduction

The fact that lipid rafts constitute a platform for various important biological processes, such as signal transduction and viral infections^1,2^, has led to increased interest in the heterogeneous distribution of sphingolipids, represented by sphingomyelin (SM), in membranes because their distribution provides pivotal information for understanding raft formation and raft-based biological events. The raft hypothesis has also stimulated physicochemical studies using artificial membrane systems comprised of SM, cholesterol (chol), and unsaturated phospholipids, such as dioleoylphosphatidylcholine (DOPC) or palmitoyloleoylphosphatidylcholine (POPC), which undergo macroscopic phase separation between raft-like liquid-ordered (L_o_) and non-raft like liquid-disordered (L_d_) domains^3–8^.

**Figure 1:**
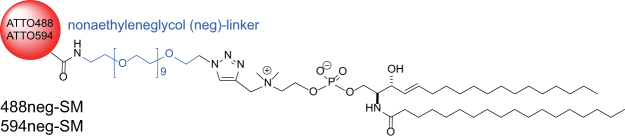
Structure of fluorescent SM analogs 488neg-SM and 594neg-SM.

Microscopic techniques have been an essential and indispensable tool for both cell and artificial membrane studies. Although a number of new microscopic technologies, such as nano-SIMS and advanced Raman microscopy, have become available for raft studies^9,10^, microscopic observations using fluorescent probes continue to be the major methodology utilized because they are applicable *in situ* with high speed and sensitivity. However, elucidation of the physicochemical and distribution properties of SM has been hampered by a lack of appropriate fluorescent probes; i.e. the currently available fluorescent SM analogs, in which fluorophores are attached to the acyl chain, favor L_d_ domains, rather than L_o_ domains, in phase-separated artificial membranes^11^. Recently, we reported the excellent fluorescent SM analogs (nonaethyleneglycol (neg)-SMs), 488neg-SM and 594neg-SM (Fig. 1), in which the hydrophilic fluorophores ATTO488 and ATTO594 are attached to the polar head of SM via a neg linker^12^. These neg-SMs exhibit partition and dynamic behaviors similar to native SM, enabling the visualization of the phase separation between the SM-rich (L_o_) and SM-poor (L_d_) domains in SM/DOPC/chol ternary giant unilamellar vesicles (GUVs). However, because fluorescence intensity depends directly on the concentration of SM in this methodology, sufficient contrast could not be obtained when the inter-domain difference in SM concentrations was not great enough.

To overcome this difficulty and enhance the contrast between the L_o_ and L_d_ domains, we propose a Förster resonance energy transfer (FRET)-based imaging using 488neg-SM and 594neg-SM that are an efficient FRET pair^13–15^. Although FRET between fluorescent lipids (Inter-lipssssid FRET) has been frequently employed in artificial membranes to monitor phase separations and phase behaviors^16–19^, to investigate formation of nano-scale domains^20–27^, and to elucidate domain sizes^28–30^, to date, application of inter-lipid FRET to imaging of lipid rafts has not been reported due to the lack of raft-philic fluorescent lipids. Our recent single-molecular study of neg-SMs has shown that the two single neg-SM molecules are temporarily colocalized more frequently in the L_o_ domain than in the L_d_ domain; the apparent colocalization lifetimes in the L_o_ and L_d_ domains are 34 ms and 12 ms, respectively^12^. Based on this result, we hypothesized that inter-lipid FRET imaging utilizing neg-SMs can confer a higher contrast ratio of L_o_-to-L_d_ domains than monochromic fluorescent imaging. In the present study, we first characterized the inter-lipid FRET of neg-SMs in SM/DOPC/chol ternary GUVs and optimized the FRET conditions applicable for raft studies. Next, we addressed the visualization of phase separation occurring in SM/POPC/chol ternary GUVs, which exhibit a lower inter-domain difference in SM distribution than SM/DOPC/chol mixtures^5^. Finally, we showed that this technique is also useful for the observation of SM-enriched domains in living cell membranes.

## Results

### Dependence of FRET intensity on membrane fluidity

**Figure 2:**
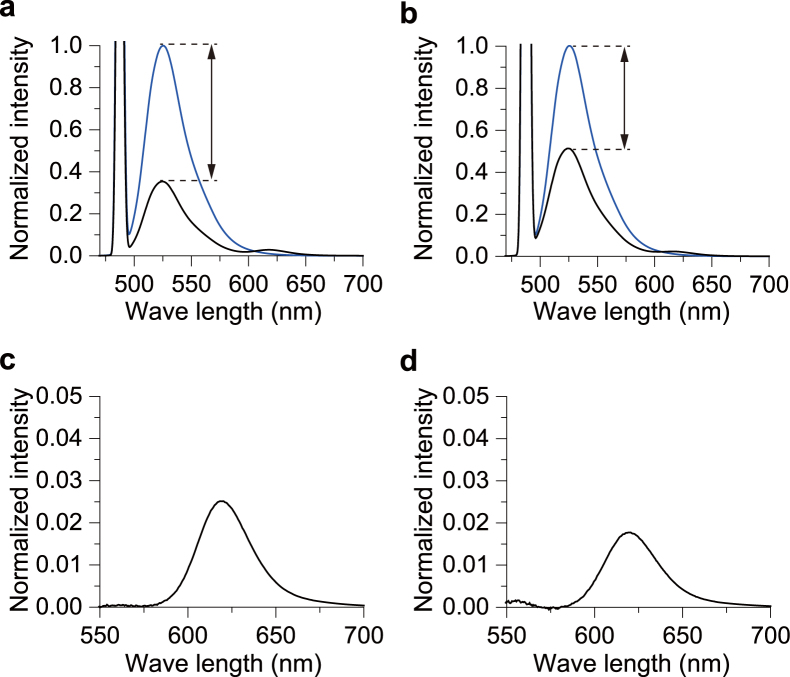
Dependence of inter-lipid FRET intensity on host membrane fluidity. Fluorescent spectra of (**a**) SM/30 mol% chol (L_o_ phase) and (**b**) DOPC (L_d_ phase) LUVs. The blue curves indicate the spectra from LUVs containing 0.2 mol% 488neg-SM (donor) only, and the black curves are from LUVs containing both 0.2 mol% 488-negSM (donor) and 0.2 mol% 594neg-SM (acceptor). The arrows show the decrease in the donor intensity due to FRET. The fluorescent intensity was normalized by the maximum intensity of the donor only spectrum (see Methods). A sharp peak at 488 nm arises from the excitation beam. (**c**,**d**) Magnified views of FRET signals of (**a**,**b**), respectively, after subtraction of the cross-talk.

Because fluorescent SM analogs are only sparsely contained in lipid bilayers that have a large excess of normal SM or other lipid molecules, we first determined whether FRET can occur between 488neg-SM and 594neg-SM in membranes. Figure 2 shows fluorescent emission spectra from SM/chol and DOPC large unilamellar vesicles (LUVs) in the presence of 0.2 mol% 488neg-SM (blue curves) or both 0.2 mol% 488neg-SM and 0.2 mol% 594neg-SM (black curves) upon excitation at 488 nm. These membranes are thought to form homogeneous L_o_ and L_d_ phases, respectively^31,32^. The spectra clearly show that SM/chol (L_o_ phase) LUVs give rise to a significantly larger decrease of donor signal at 528 nm and a higher FRET intensity at 620 nm than DOPC (L_d_ phase) LUVs, although the concentration of neg-SMs is identical between the two membrane systems. This indicates that lower fluidity leads to higher FRET intensity under the same concentration of neg-SMs, thus suggesting its potential applicability for inter-lipid FRET imaging. We further confirmed that these results hold true for giant unilamellar vesicles (GUVs) (Fig. S1).

### Characterization of inter-lipid FRET between neg-SMs

**Figure 3:**
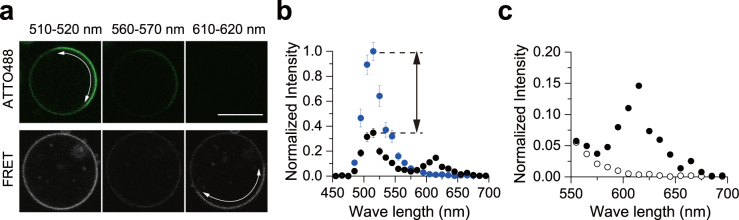
Characterization of inter-lipid FRET between 488neg-SM and 594neg-SM in phase-segregated membranes. (**a**) Confocal fluorescence micrographs at the equatorial plane of SM/DOPC/chol (in a mole ratio of 1:1:0.5) ternary GUVs in the presence of 488neg-SM only (top) or both 488neg- and 594neg-SMs (bottom). Excitation wave length was 473 nm, and the detection wave lengths (*λ*_det_) are detailed above the panels. The SM-rich (L_o_) regions are indicated by the curved arrows and the bar indicates 10 μm. The brightness was enhanced for clearer visualization. (**b**) Fluorescent intensities of L_o_ domains of GUVs containing both 488neg- and 594neg-SMs (black circles) or 488neg-SM only (blue circles). The data were collected as a function of *λ*_det_ every 10 nm step and averaged in 21–27 GUVs. The error bars represent standard error. The arrows show the decrease in the donor intensity due to FRET. The fluorescent intensity was normalized by the maximum intensity of the blue circle. (**c**) Magnification of FRET signals (filled circles) and cross-talk (open circles). The error bars were removed for clarity.

We next optimized the experimental conditions for detecting pure FRET signals in GUVs. We first prepared SM/DOPC/chol ternary GUVs containing 0.2 mol% 488neg-SM or both 0.2 mol% 488neg-SM and 0.2 mol% 594neg-SM, and scanned the detection wave lengths (*λ*_det_) from 450 to 700 nm upon excitation at 473 nm. As shown in Fig. 3a, the FRET signal was clearly detected at 610 nm–620 nm (Fig. 3b,c). In subsequent FRET imaging experiments, we adopted *λ*_det_ = 610–620 nm to obtain pure FRET signals.

**Figure 4:**
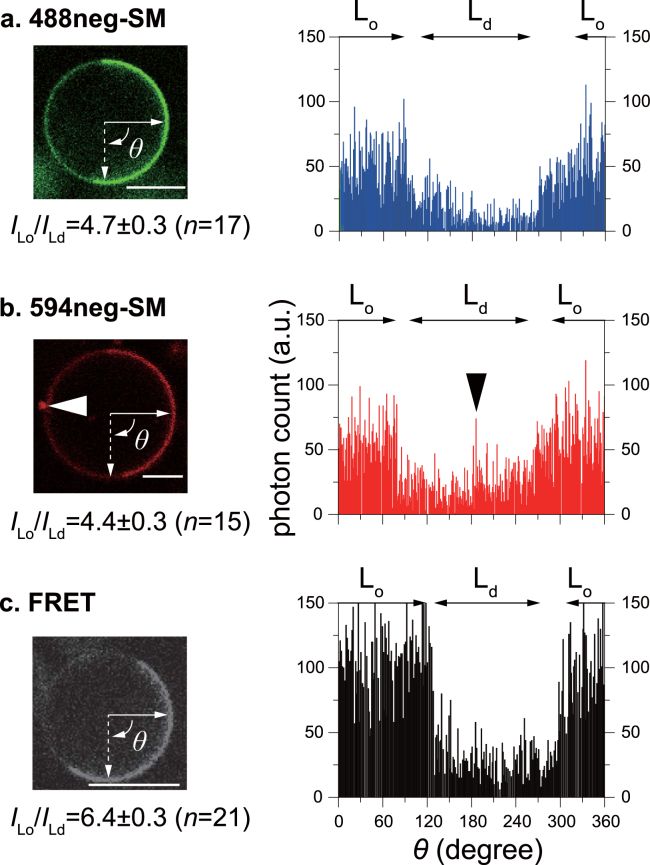
Confocal fluorescence micrographs at the equatorial planes of GUVs comprised of SM/DOPC/chol (in a mole ratio of 1:1:0.5; left-hand panels) and the intensity profiles along the edge of the GUVs (right-hand panels). Bars indicate 10 μm. Monochromic detection of GUVs containing 0.2 mol% 488neg-SM (**a**) or 594neg-SM (**b**,**c**) FRET detection of GUVs containing 0.2 mol% 488neg-SM and 0.2 mol% 594neg-SM. The intensity ratios between the L_o_ and L_d_ domains (*I*_Lo_/*I*_Ld_), which were obtained by averaging the data of 15–21 GUVs (the numbers of GUVs are given in parentheses), are shown below the corresponding micrographs (the intensity data are presented in Table S1). A prominent signal in the L_d_ region of (**b**) was derived from a debris on the edge of the GUV (indicated by arrow) and was excluded in the intensity analysis. The errors indicate standard error (SE).

To confirm that the inter-lipid FRET technique provides higher contrast, we next compared the intensity ratio of SM-rich (L_o_) to SM-poor (L_d_) domains (*I*_Lo_/*I*_Ld_) between FRET and conventional monochromic images. Figure 4a,b (left-hand panels) show confocal monochromic images at the equatorial planes of SM/DOPC/chol GUVs containing 0.2 mol% 488neg-SM and 594neg-SM, respectively. Based on these results, we plotted the fluorescent intensities along the edges of the GUVs as a function of *θ* (Fig. 4a,b, right-hand panels). Applying this intensity analysis to 17 and 15 GUVs, respectively, we estimated the average ratio of fluorescent intensity of the L_o_ domain to be 4.55-fold higher than that of the L_d_ domain; *I*_Lo_/*I*_Ld_ = 4.7 ± 0.3 for 488neg-SM and *I*_Lo_/*I*_Ld_ = 4.4 ± 0.3 for 594neg-SM. These results are in line with previous intensity analyses although lipid composition was slightly different^12^. Figure 4c shows the confocal FRET image at the equatorial plane of the GUV and its intensity analysis, which revealed that the L_o_ domain is 5.9 ± 0.2–fold brighter than the L_d_ domain. These results demonstrate that FRET imaging improves the inter-domain contrast by 41% (=[6.4–4.55]/4.55) compared to monochromic imaging in this system, thus confirming that inter-lipid FRET between neg-SMs can emphasize L_o_ domains.

**Figure 5:**
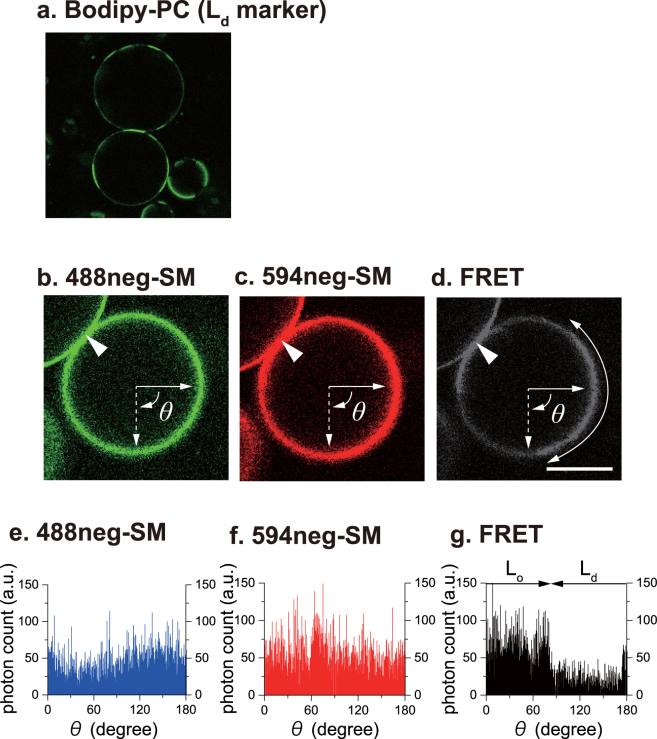
FRET-enhanced intensity ratio between the L_o_ and L_d_ domains. Confocal fluorescent images of SM/POPC/chol (in a mole ratio of 1:1.2:0.5) GUVs in the presence of 0.2 mol% Bodipy-PC (**a**) and neg-SMs (**b**–**d**). The micrograph of GUVs (**a**) was different from those of (**b**–**d**), while the images (**b**–**d**) were obtained from the same GUVs. The L_o_ domains are indicated by curved arrows (**d**). The bar indicates 20 μm. (**e**–**g**) show the intensity profiles along the dashed lines in (**b**–**d**), respectively. Intensity profiles of (**b**–**d**) along the edge of the GUV were plotted in (**e**–**g**), respectively, from *θ* = 0° to 180°. The merged regions of the two GUVs (indicated by arrowheads in **b**–**d**) are not included in the angle range.

Next, we applied inter-lipid FRET imaging to phase-segregated SM/POPC/chol ternary GUVs. In these GUVs, the difference in SM distribution between the L_o_/L_d_ domains is smaller than in the aforementioned SM/DOPC/chol ternary system^5^ and thus domain separation is indistinguishable using monochromic imaging, especially in the vicinity of the miscible-immiscible phase boundary. Figure 5a shows a confocal monochromic fluorescence micrograph of SM/POPC/chol GUVs in the presence of Bodipy-PC, which is an L_d_ phase marker in phase-separated GUVs^33^. This micrograph demonstrates that the ternary GUV undergoes phase separation under the given conditions. In contrast, the monochromic fluorescent micrographs of 488neg-SM and 594neg-SM barely detect the phase separation (Fig. 5b,c), because of the smaller neg-SM distributional differences between these two domains. Figure 5d shows the inter-lipid FRET image of the same GUV presented in Fig. 5b,c. Notably, FRET imaging provided sufficient contrast between the two phases, thus enabling visualization of phase segregation. Here we confirmed that the regions showing higher and lower FRET intensity in Fig. 5d correspond to the L_o_ and L_d_ domains, respectively, by measuring diffusion coefficients using fluorescent correlation spectroscopy (Fig. S2). In order to compare the contrast between the two imaging techniques, the intensity profiles along the edge of the GUV are shown in Fig. 5e–g. Apparently, inter-lipid FRET imaging induced a higher intensity ratio than monochromic imaging.

### Application of inter-lipid FRET imaging in living cell membranes

**Figure 6:**
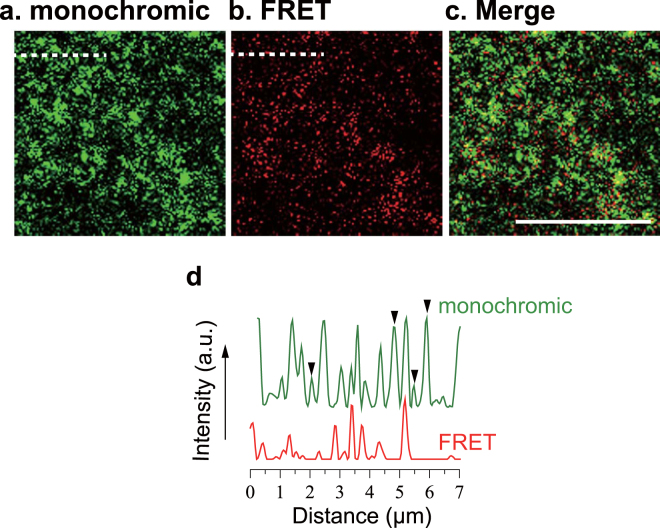
Single-cell confocal (**a**) monochromic, (**b**) FRET, and (**c**) their merged images of the apical membrane of cultured epithelial (EpH4) cells labeled with 488neg-SM and 594neg-SM. These images show the same region. The bar indicates 10 μm. Green and red profiles in (**d**) represent the fluorescent intensity along the dashed lines in (**a**) and (**b**), respectively. Arrows indicate peaks with intense monochromic signals, but weak or no FRET, and an asterisk indicates a FRET signal with weak monochromic peak.

Finally, we examined whether inter-lipid FRET imaging is useful for visualizing SM-rich membrane micro-domains in living cells. Prior to applying inter-lipid FRET imaging to living cells, we confirmed that Hanks’ balanced salt solution (HBSS), in which the cell observation was performed, did not affect inter-lipid FRET intensities in artificial membranes (Fig. S3). Previous studies have shown that SM is enriched in the apical membrane of epithelial cells^34,35^. When 488neg-SM and 594neg-SM were incorporated into the apical membrane of cultured epithelial (EpH4) cells, they mainly colocalized in the discrete micro-domains of the apical membrane (Fig. 6a). However, it is unlikely that the entire regions visualized by monochromic imaging constitute SM-rich micro-domains, because exogenously added fluorescent SMs can be incidentally partitioned at a high concentration into membrane regions where SMs are relatively sparse. Therefore, using inter-lipid FRET imaging, we tried to visualize the precise localization of SM-rich domains in the apical membrane. As shown in Fig. 6b, inter-lipid FRET signals were observed only in partial regions of the membrane and the stained area is much smaller in comparison with Fig. 6a. For further detailed analysis, monochromic and FRET images were merged (Fig. 6c), and line intensity profiles were compared between monochromic and FRET imaging (Fig. 6d). Interestingly, several monochromic and FRET signals were not regionally superposed (indicated by arrowheads and asterisk in Fig. 6d). Because FRET occurs only when both 488neg-SM and 594neg-SM are near each other (within several nanometers; see Discussion), it is reasonable to assume that SM-condensed micro-domains can be emphasized more accurately by inter-lipid FRET imaging.

## Discussion

In this study, we applied inter-lipid FRET for the imaging of L_o_ and L_d_ phase separation and demonstrated that FRET imaging can emphasize L_o_ domains by enhancing the inter-domain contrast better than conventional monochromic imaging. In fact, inter-lipid FRET imaging enabled the visualization of the phase separation of POPC/SM/cholesterol GUVs, in which the difference in SM concentration is so slight that monochromic imaging could barely detect phase separation (Fig. 5). To the best of our knowledge, this is the first observation of inter-lipid FRET imaging using raft-specific fluorescent probes.

Why does FRET imaging exhibit higher inter-domain contrast than monochromic imaging? Our recent single fluorescent molecule tracking experiments revealed that the apparent colocalization lifetime of neg-SMs is 2.8-fold greater in L_o_ (34 ms) than in L_d_ planar membranes (12 ms)^12^. The apparent colocalization lifetime is the duration during which two fluorescent molecules colocalize within the diffraction limit (*Φ*~ 240 nm) of the laser, and has also been reported to correlate with FRET lifetime^36^. Thus, the L_o_ domain with a longer colocalization lifetime should produce inter-lipid FRET more effectively than the L_d_ domain, even if the concentration of neg-SMs is identical between the two domains. In effect, under the same concentration of neg-SMs, the L_o_ membrane exhibits significantly higher FRET intensity than the L_d_ membrane (Fig. 2). We further found that the FRET intensity of dipalmitoylphosphatidylcholine (DPPC)/chol LUVs (Fig. S4) was slightly smaller than that of SM/Chol (Fig. 2), although DPPC/chol also forms the L_o_ domain. This can be accounted for by lower domain-stability of PC/chol than SM/chol due to weaker inter-lipid interactions^37^. These results indicate that a greater inter-lipid interaction in the L_o_ domains and the resulting higher cluster formation of SM elongate the colocalization lifetime and promote the energy transfer between ATTO488 and ATTO594, consequently leading to a higher inter-domain contrast in inter-lipid FRET than in monochromic imaging.

In this study, the acquisition time for the inter-lipid FRET imaging (40 μs/pix) was set longer than that for the monochromic imaging (8 μs/pix) (see § Methods) because of the weaker FRET intensity than the emission from directly excited fluorophore (Fig. 2). Hence, in terms of the time-resolution, monochromic imaging is superior to inter-lipid FRET. However, because both ATTO488 and ATTO594 are much more tolerant to laser exposure than other fluorescent dyes, it is possible to enhance the inter-lipid FRET intensity between neg-SMs by using stronger laser excitations, which would extend the application of inter-lipid FRET to high-speed and time-laps imaging.

We further applied inter-lipid FRET imaging to living cell membranes (Fig. 6). Considering that the Förster distance between ATTO488 and ATTO594 is 5.6 nm^38^, the presence of FRET signals suggests that both FRET-pair molecules, 488neg-SM and 594neg-SM, are highly condensed in small regions such as an SM-assembly or cluster. The signal patterns in Fig. 6 can be classified as strong FRET and monochromic signals, strong FRET but weak monochromic signals (asterisk in Fig. 6d), and weak or no FRET with strong monochromic signals (arrowheads in Fig. 6d). The first and second patterns indicate the occurrence of inter-lipid FRET, and in particular, the second pattern can be explained by the occurrence of FRET-quenching of the donor (488neg-SM) signals by a larger amount of surrounding acceptor (594neg-SM) molecules. Of note is the third pattern, in which membrane regions have strong monochromic signals, but weak or no FRET signals (arrowheads in Fig. 6d). A possible explanation for this is that either one of the two fluorescent SMs (in this case, the donor 488neg-SM) was incidentally localized to those regions as a result of the exogenous addition, and/or exuded from SM-rich domains to those regions. If this is the case, inter-lipid FRET can exclude membrane regions into which the fluorescent SMs were incidentally spotted and/or oozed. Another possibility is that the SM molecules in those membrane regions are not packed tightly enough to form the SM-condensed assemblages or clusters prerequisite for FRET occurrence. In either case, inter-lipid FRET imaging can emphasize SM-concentrated assemblages and clusters (such as raft domains) in living cell membranes, because FRET occurs only when 488neg-SM and 594neg-SM are near each other (within the nanometer range). In this context, the inter-lipid FRET imaging technique using neg-SMs paves the way to emphatically visualize SM-enriched domains both in artificial and biological membranes.

Recent studies have demonstrated that lipid rafts in real cell membranes are fragile and transient domains^39–42^, and thus it is unlikely that L_o_ phase present in artificial membranes does completely reproduce modestly ordered raft structures in living cell membranes^1,43–45^. In fact, the formation of nanometer-sized domains is predicted to occur in cell membranes and detection of these domains is an important question in the field of raft studies^45^. In addition, we have reported that SM in cell membranes forms nano-clusters (smaller in size than the resolution of optical microscopes) more favorably than other glycerophospholipids^10^. FRET has been the most widely used technique for obtaining structural information at the nanometer scale. However, to data, SM nano-domain formation in biomembranes has not been fully characterized because of the lack of appropriate fluorescent probes that reproduce the dynamic and partition behaviors of SM in membranes. Therefore, the combination of neg-SMs and inter-lipid FRET imaging constitutes a promising tool not merely for visualizing SM-rich domains, but also for obtaining nano-scopic information on lipid rafts in live cell membranes.

Another important feature of the present method is the possibility of detecting lipid-lipid interactions in membranes. Although it is generally believed that molecular interactions between sphingolipids (such as SM) and chol are essential for raft formation, we have recently proposed that the formation of intermolecular hydrogen bonds between the amide groups in adjacent SM molecules is more important for the raft formation^37,46,47^. As mentioned above, a higher L_o_-L_d_ contrast in inter-lipid FRET can be accounted for by a stronger inter-lipid interaction and the resulting elongated colocalization in SM-enriched domains. However, it is still very difficult or impossible to directly detect inter-lipid interactions in live cell membranes due to the lack of appropriate methodology. Accordingly, our technique will also provide unique and invaluable information on lipid-lipid interactions in live cells and shed new light on their importance in raft formation.

## Methods

### Materials

Brain SM and DOPC were purchased from Avanti Polar Lipids (Alabaster, AL, USA), and chol was purchased from Sigma Aldrich (St. Louis, MO, USA). Stearoyl-SM (SSM) was purified from brain SM using a SPD-M10A HPLC (Shimadzu, Shizuoka, Japan) with a 5C18-MS-II C18 reverse-phase column (Nacalai Tesque, Kyoto, Japan) as previously reported^48^, with slight modification. Purity was determined by thin layer chromatography with Silica gel 60 F_254_ HX44026754 (Merck, Darmstadt, Germany). 2-(4,4-difluoro-5,7-dimethyl-4-bora-3a,4a-diaza-s-indacene-3-dodecanoyl)-1-hexadecanoyl-sn-glycero-3-phosphocholine (Bodipy-PC) was purchased from Thermo Fisher Scientific Inc. (Waltham, MA, USA). 488neg-SM and 594neg-SM, labeled with ATTO488 and ATTO594 (ATTO-TEC GmbH, Siegen, Germany), were synthesized as previously described^12^.

### Fluorescent spectroscopy

Large unilamellar vesicles (LUVs) of pure DOPC or SSM/30 mol% chol containing both 488neg-SM and 594neg-SM were prepared in order to measure fluorescent spectroscopy. Pure DOPC (100 μmol) or SSM/30 mol% chol (total 100 μmol) were dissolved in chloroform/methanol (4:1 v/v) in the presence of 0.2 mol% 488neg-SM or both 0.2 mol% 488neg-SM and 0.2 mol% 594neg-SM, and then dried under an N_2_ flow. The remaining organic solvent was completely removed in vacuo for >12 h. The dried lipid films were suspended in 1 mL of Milli-Q water at 40 °C for pure DOPC and 70 °C for SM/chol with intermittent vortexing to form MLVs. The MLV suspensions were extruded through a two-ply membrane filter with a pore size of *Φ* = 100 nm (GE Healthcare UK Ltd., Buckinghamshire, UK) 19 times using an Avanti mini-extruder (Avanti Polar Lipid Inc., Alabaster, AL, USA) to form LUVs. The LUV samples were suspended in 2 mL of Milli-Q water and equilibrated for more than 30 min at 25 °C prior to measurements. Excitation was applied at 488 nm and emission spectra were obtained from 450 to 700 nm every 5 nm step using a FP-8300 spectrofluorometer (JASCO Corp., Tokyo, Japan). The temperature was maintained at 25 °C with a CTU-100 water circulator (JASCO Corp., Tokyo, Japan). The spectra were standardized according to lipid concentrations determined by phosphorus assay Phospholipids C (Wako Pure Chemicals Industries Ltd. Osaka, Japan). Then, the emission spectra were normalized by the maximum intensity of the sample containing only 488neg-SM. To avoid the cross-talk and gain pure FRET signal, we subtracted the emission spectrum of sample containing 488neg-SM from that containing both of 488neg- and 594neg-SMs after the maximum emission intensity of the 488neg-SM-containing sample was reduced to be equal to that of the sample containing both neg-SMs. Emission of 594neg-SM under *λ*_ex_ = 488 nm irradiation was negligibly small (less than 4% of FRET intensity).

### Giant unilamellar vesicle (GUV) preparation

GUVs of ternary lipid mixtures were prepared by electroformation^49^. Briefly, a mixture of SSM/DOPC/chol (1:1:0.5) or SSM/POPC/chol (1:1.2:0.5) containing fluorescent lipids (0.2 mol% 488neg-SM, 0.2 mol% 594neg-SM, both of them, or 0.2 mol% Bodipy-PC) was dissolved in chloroform-methanol to obtain a sample solution (1 mg/mL). A 7 μL aliquot of the sample solution was spread on the electrode surface (platinum wires, *Φ = *100 μm; Nilaco, Tokyo, Japan), and the organic solvent was removed in vacuo for 24 h. The two electrodes coated with the thin lipid film were attached on both sides of a square-shaped rubber seal (1-mm thick, with a square window of 15 × 15 mm), containing Milli-Q water (Simplicity UV, Merck; Darmstadt, Germany) and then sandwiched between two cover glasses (24 mm × 60 mm, 0.12–0.17 mm thickness; Matsunami, Shizuoka, Japan). This chamber was placed on a temperature-controlled sample stage (Tokai Hit, Shizuoka, Japan) on the confocal fluorescence microscope, and then a low-frequency (10 Hz) sinusoidal current (10 Vpp) was applied using an Agilent function generator (Model 33120 A; Santa Clara, CA, USA), at 55 °C for 60 min (the temperature was calibrated with a K-type thermocouple sensor AD-5602A; Sanyo Industries Ltd., Tokyo, Japan). The GUVs formed in this chamber were equilibrated at 25 °C for 15 min for subsequent microscopic observation.

### Confocal laser-scanning fluorescence microscopy and intensity analysis

Confocal laser-scanning fluorescence microscopy was performed at 25 °C on an Olympus FV1000-D IX81 microscope (Tokyo, Japan) using an objective lens with a long working distance (LUCPLFLN 60×, N.A. = 0.7). The images were obtained with the acquisition software FV10-ASW4.2.

To obtain fluorescent intensities (Fig. 2), we applied excitation laser at *λ*_ex_ = 473 nm (17.8 μW) and detection wave length was scanned from 450–700 nm every 10 nm step. We used laser scanning rates of 2 μs/pix and 4 μs/pix (1024 pix × 1024 pix) for the intensity analysis of the L_o_ and L_d_ domains, respectively. The fluorescent intensities were normalized by the maximum emission from the sample containing 488neg-SM.

For monochromic fluorescence observation, we applied the excitation lasers at *λ*_ex_ = 473 nm (8.9 μW) and 559 nm (6.0 μW), and the emissions were detected between 485–540 nm and 570–670 nm for 488neg-SM and 594neg-SM, respectively. For FRET imaging, we detected the FRET signal at 610–620 nm. We used laser scanning rates of 8 μs/pix and 40 μs/pix to obtain monochromic and FRET images (1024 pix × 1024 pix), respectively.

Intensity ratios between the L_o_ and L_d_ domains were estimated in accordance with previous methods^12^. Briefly, we obtained the fluorescent intensities along the edges of the GUVs with FV10-ASW4.2 software and analyzed it using OriginPro 2015J (Lightstone, Tokyo, Japan). For the analysis we used the center region of the domains (indicated by arrows in Fig. 4) and excluded ambiguous phase boundary regions. Then, we calculated the intensity ratio between the L_o_ and L_d_ domains (*I*_Lo_/*I*_Ld_) for each GUV after subtracting background intensity and cross-talk. Appling this analysis for 15~21 GUVs, we determined the average ratio of fluorescent intensity between the two domains. In the present study, the contrast was defined by the intensity ratio between the two domains because the intensity ratio does not depend on exposure time.

### Cell culturing and *in vivo* staining

EpH4 cells (generously provided by Dr. E. Reichmann, Institute Suisse de Recherches, Lausanne, Switzerland) were grown in Dulbecco’s modified Eagle’s medium supplemented with 10% fetal calf serum. Cells were plated onto glass-base dishes (Iwaki Glass, Tokyo, Japan) and grown to confluency. 488neg-SM and 594neg-SM were incorporated into the apical membrane of EpH4 cells by incubations with 1 µM (final concentration) fluorescent lipids in Hanks’ Balanced Salt solution (HBSS) on ice for 5 min. Cells were washed three times with HBSS, then subjected to the fluorescence imaging. Fluorescence imaging was performed using a 63× oil-immersion objective on an inverted microscope LSM700 (Carl Zeiss MicroImaging; Thornwood, New York) interfaced to a laser-scanning confocal microscope. FRET images were acquired on a PC using ZEN2012 software (LSM700; Carl Zeiss MicroImaging).

## Electronic supplementary material

Supplementary Information
