# Supplementary material for: Emphatic visualization of sphingomyelin-rich domains by inter-lipid FRET imaging using fluorescent sphingomyelins
Source: Sci Rep. 2017 Dec 1;7:16801. doi: 10.1038/s41598-017-16361-x (PMC5711942; doi:10.1038/s41598-017-16361-x)
Supplement: Supplementary file 1 — Supplementary Information [file 41598_2017_16361_MOESM1_ESM.pdf]

Supplementary information:

Emphatic visualization of sphingomyelin-rich domains by inter-lipid FRET imaging using fluorescent sphingomyelins

Masanao Kinoshita[1,2,3*], Hikaru Ano[1,2], Michio Murata[1,2,4], Kenta Shigetomi[5], Junichi Ikenouchi[5] & Nobuaki Matsumori[1,3,4*]

[1] JST-ERATO Lipid Active Structure Project, Osaka University, 1-1 Machikaneyama, Toyonaka, Osaka, 560-0043, Japan.

[2] Project Research Center for Fundamental Science, Osaka University, 1-1 Machikaneyama, Toyonaka, Osaka, 560-0043, Japan.

[3] Department of Chemistry, Faculty of Science, Kyushu University, 744 Motooka, Nishi-ku, Fukuoka, 819-0395, Japan.

[4] Department of Chemistry, Graduate School of Science, Osaka University, 1-1 Machikaneyama, Toyonaka, Osaka, 560-0043, Japan.

[5] Department of Biology, Faculty of Science, Kyushu University, 744 Motooka, Nishi-ku, Fukuoka, 819-0395, Japan.

[*]Correspondence and requests for materials should be addressed to N. M. (email: matsmori@chem.kyushu-univ.jp) and M. K. (email: kinoshi@chem.kyushu-univ.jp).

Supplementary Methods

Confocal fluorescence microscopy and intensity analysis. SSM/30 mol% chol, DPPC/30 mol% chol and pure DOPC GUVs were prepared following the method described in the main text and observed with the same microscope used in the main text. In fluorescent spectroscopy, we applied excitation laser at λ_{ex}= 473 nm (17.9 μW) and detection wave length was scanned from 450-700 nm every 10 nm step. We used laser scanning rates of 2 μs/pix (1024 pix×1024 pix). The obtained spectra were analyzed as well as those in the main text.

Fluorescent correlation spectroscopy (FCS) measurements. FCS measurements of GUV surfaces were performed at 25˚C with the same microscope used to obtain the confocal fluorescence images, with an oil immersion apochromat objective lens (Olympus PLAPON60XO, 60x, N.A.=1.4), following the protocol published previously[1,2]. The diffusion coefficient, *D*, was obtained by fitting the autocorrelation function of the time-dependent changes of the signal intensities of fluorescent probe molecules in diffraction-limited spots, $G(\tau)$, with the following equation for two-dimensional simple-Brownian diffusion:

$$\mathrm{G}(\tau) = \frac{1}{N}\left\{\frac{1}{1+\left(\frac{4D\tau}{W_0^2}\right)}\right\} \quad (1)$$

where *N* is the average number of fluorescent particles in the detection area, W_0 is the beam radius in the focal plane (W_0=0.16 μm for excitation wavelengths of 559 nm), *D* is the diffusion coefficient, and τ is the delay time. The FCS measurements were performed in GUVs containing 0.008 mol% 488neg-SSM, 594neg-SSM.

References

[1] Korlach, J., P. Schwille, & W.W. Webb. Characterization of lipid bilayer phases by confocal microscopy and fluorescence correlation spectroscopy. *Proc. Natl. Acad. Sci. U.S.A.* 96, 8461-8466

(1999).

[2] Kahya, N., D. Scherfeld, K. Bacia, B. Poolman, & P. Schwille. Probing lipid mobility of raft-exhibiting model membranes by fluorescence correlation spectroscopy. *J. Biol. Chem.* **278**, 28109-28115 (2003).

Supplementary Figures

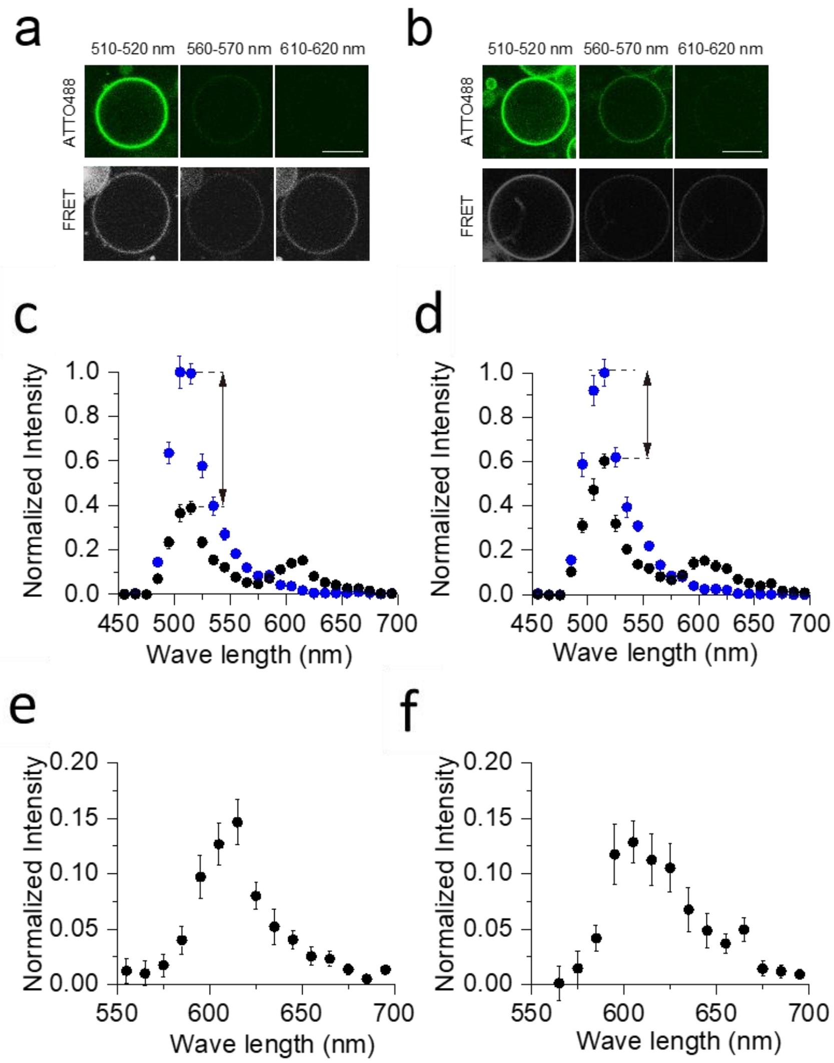

Figure S1. Influence of membrane properties on FRET intensity in GUVs. Confocal fluorescent micrographs of (**a**) SM/30 mol% chol (L_o phase) and (**b**) DOPC (L_d phase) GUVs. These GUVs contain 488neg-SM (donor) only (top) and 488neg-SM (donor)/594ng-SM (acceptor) (bottom). The brightness was enhanced for clearer visualization. The detection wave length was depicted in the figure. (**c**, **d**) Averaged fluorescent spectra obtained in 20-24 GUVs containing donor only (blue circles) and donor/acceptor (black circles). Lipid compositions of (**c**, **d**) correspond to (**a**, **b**), respectively. The arrows show the decrease in the donor intensity due to FRET. The fluorescent intensity was normalized by the intensity of donor only (blue curve). (**e**, **f**) Magnified views of FRET signals in (**c**, **d**), respectively, after subtraction of the cross-talk.

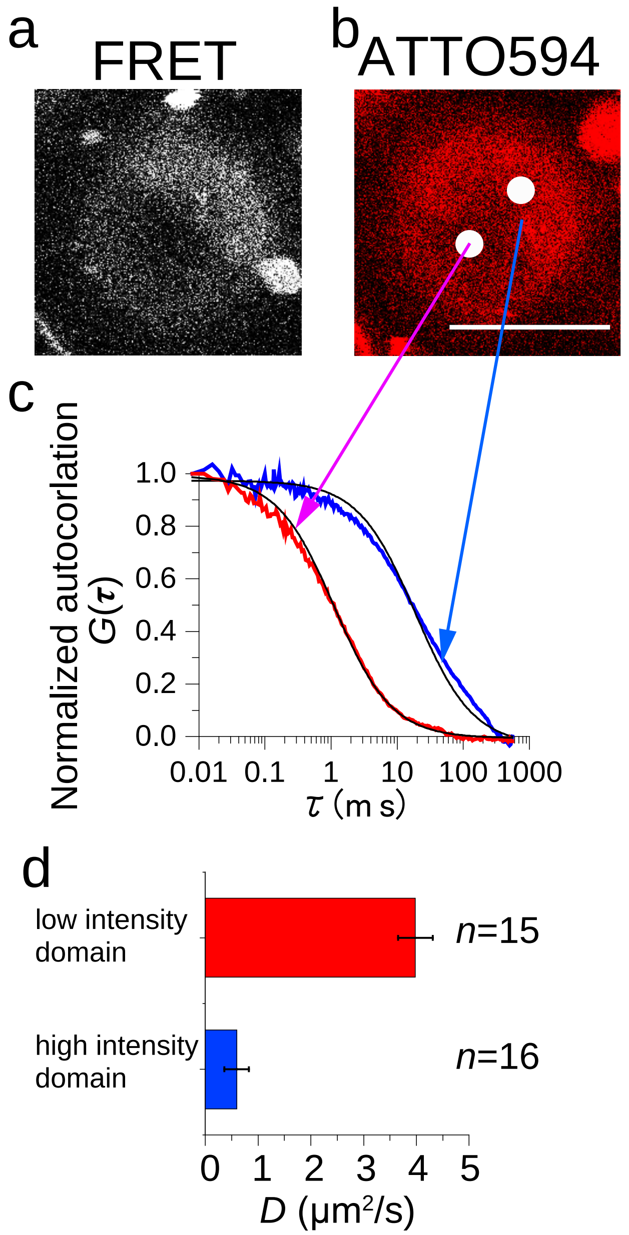

Figure S2. Phase-identification by FCS measurements. (**a**) FRET and (**b**) monochromic images of SM/POPC/chol (1:1.2:0.5 by moles) GUV surface. The brightness was enhanced for clearer visualization. A bar indicates 20 μm. (**c**) FCS curves obtained in the brighter and darker regions. The spots show the laser locations (the laser size was expanded 5× to show their location clearly). (**d**) Diffusion coefficients estimated by fitting the FCS curves to theoretical function Eq. (1). This confirmed that the brighter and darker regions correspond to the L_o and L_d phases, respectively.

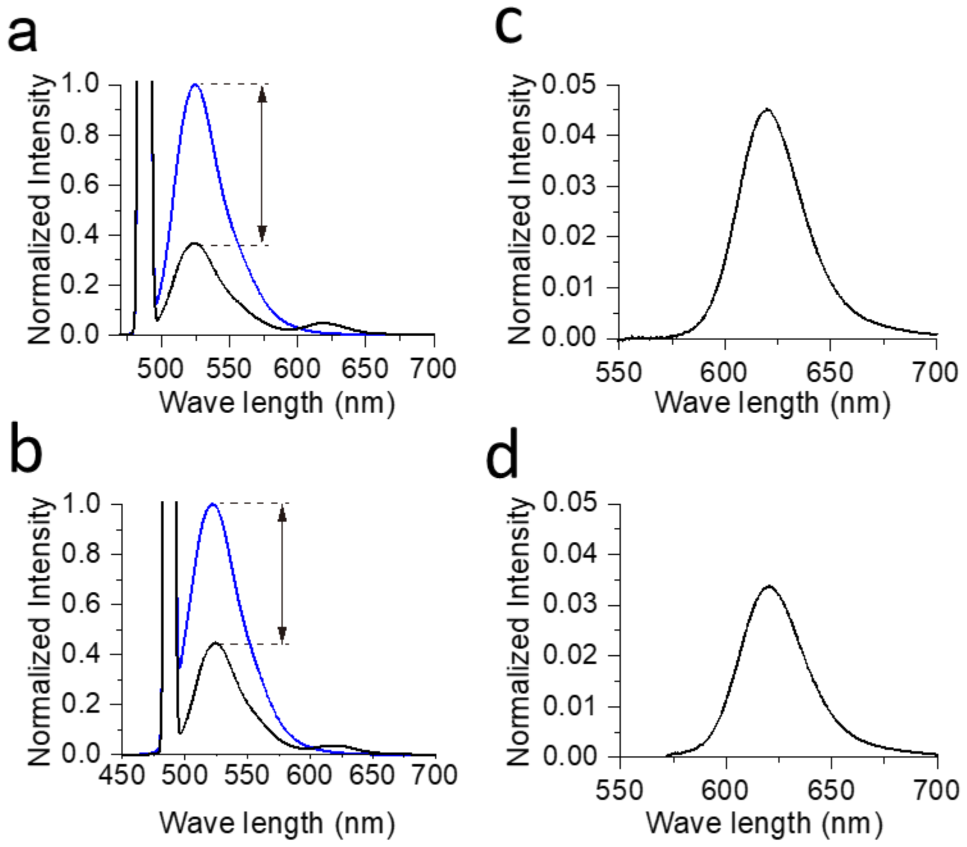

Figure S3. Influence of HBSS buffer on the FRET intensity. Fluorescent spectra of (**a**) SM/30 mol% chol (L_o phase) and (**b**) DOPC LUVs (L_d phase), respectively, in HBSS buffer. These LUVs contain 488neg-SM (donor) only (blue curve) or both 488neg- and 594neg-SMs (black curve). The arrows show the decrease in the donor intensity due to FRET. The fluorescent intensity was normalized by the maximum intensity of donor only (blue curve). A sharp peak at 488 nm arises from the excitation beam. (**c**, **d**) Magnified views of FRET signals in (**a**, **b**), respectively, after subtraction of the cross-talk.

.

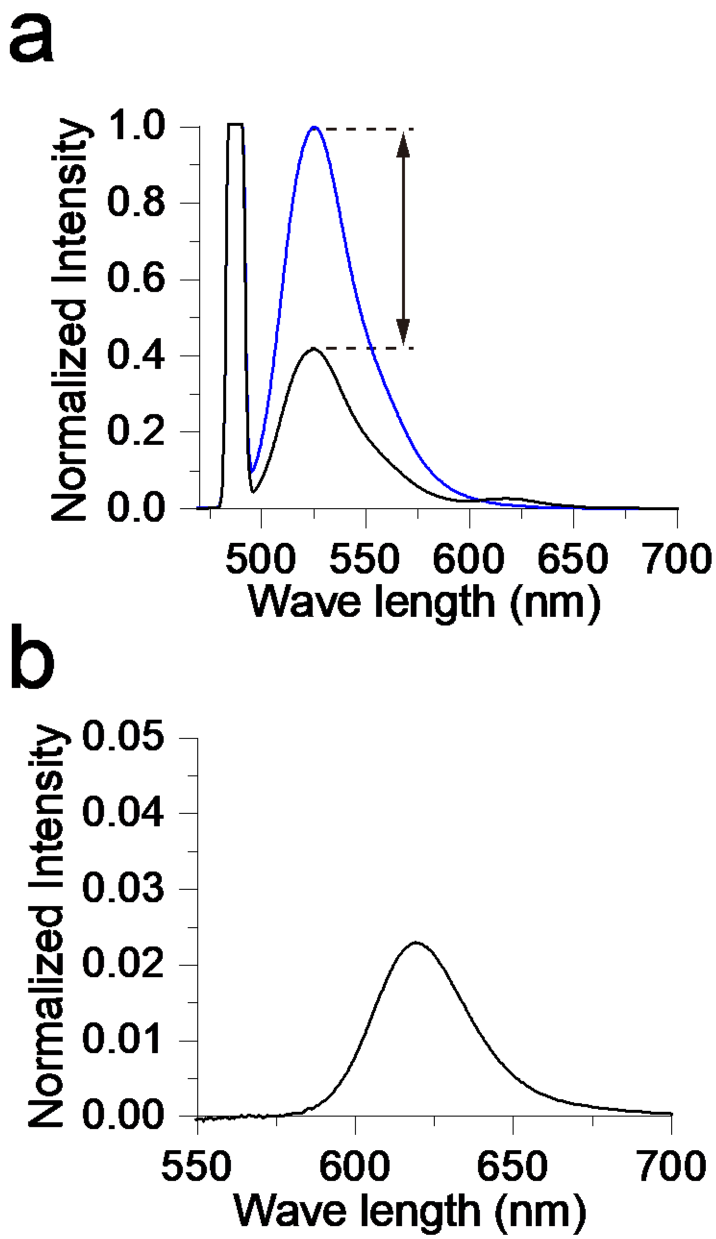

Figure S4. Evaluation of Inter-lipid FRET in the L_o membrane comprising of dipalmitoylphosphatidylcholine (DPPC)/chol. (**a**) Fluorescent spectra of DPPC/30 mol% chol (L_o phase) LUVs. The sample contain the donor only (blue curve) and the donor/acceptor (black curve). The arrow shows the decrease in the donor intensity due to FRET. The fluorescent intensity was normalized by the maximum intensity of donor only (blue curve). A sharp peak at 488 nm corresponds to intensity of excitation beam. (**b**) Magnified views of FRET signals in (**a**) after subtraction of the cross-talk.

Supplementary Table

Table S1. Photon counting data (a.u.) for Figure 4.

Fig. 4a			Fig. 4b			Fig. 4c		
L_o domain	L_d domain	ratio	L_o domain	L_d domain	ratio	L_o domain	L_d domain	ratio
94.1	24.1	3.90	57.1	15.1	3.78	43.9	7.73	5.68
139.1	34.1	4.08	59.1	15.1	3.91	55.9	9.11	6.14
74.1	19.1	3.88	79.1	16.1	4.91	49.9	6.35	7.86
94.1	14.1	6.67	94.1	34.1	2.76	53.9	6.35	8.49
84.1	14.1	5.96	84.1	29.1	2.89	38.9	7.27	5.35
69.1	21.1	3.27	69.1	19.1	3.62	38.9	8.19	4.75
109.1	17.1	6.38	49.1	15.1	3.25	53.9	9.11	5.92
119.1	19.1	6.24	46.1	9.1	5.07	63.9	12.79	5.00
84.1	19.1	4.40	49.1	9.1	5.40	46.9	7.27	6.45
134.1	29.1	4.61	57.1	12.1	4.72	56.9	8.65	6.58
59.1	14.1	4.19	42.1	6.1	6.90	43.9	7.27	6.04
54.1	14.1	3.84	57.1	12.1	4.72	69.9	7.27	9.62
124.1	24.1	5.15	71.1	13.1	5.43	40.9	5.89	6.95
101.1	29.1	3.47	52.1	11.1	4.69	60.9	10.03	6.07
119.1	24.1	4.94	89.1	27.1	3.29	56.4	9.57	5.89
129.1	31.1	4.15				16.9	2.67	6.33
94.1	19.1	4.93				37.9	5.89	6.44
						40.9	6.53	6.26
						45.9	8.65	5.31
						38.9	5.43	7.17
						33.9	5.89	5.76
Avg.		4.7			4.4			6.4
S.E.		0.3			0.3			0.3
